# The impact of smart city construction (SCC) on pollution emissions (PE): evidence from China

**DOI:** 10.1038/s41598-024-57138-3

**Published:** 2024-03-19

**Authors:** GuoWei Zhang, XianMin Sun, Shen Zhong

**Affiliations:** 1 School of Economics, Harbin University of Commerce, Harbin, 150028 China; 2 School of Finance, Harbin University of Commerce, Harbin, 150028 China

**Keywords:** Smart city construction, Time-varying DID, Volume of sewage discharge, Sulfur dioxide emissions, Emissions of fumes and dust, Pollution emissions, Environmental impact, Environmental impact, Sustainability

## Abstract

Based on panel data from 210 prefecture-level cities in China from 2003 to 2021, this study employs the Time-Varying Differences-in-Differences (Time-Varying DID) approach to systematically examine the impact of smart city construction on pollution emissions and its underlying mechanisms. Additionally, the Propensity Score Matching–Differences-in-Differences method is employed for further validation. The research findings indicate that Smart City Construction (SCC) significantly reduces urban Volume of Sewage Discharge (VSD), sulfur dioxide emissions (SO_2_), and Emissions of Fumes and Dust (EFD), thereby mitigating pollution emissions (PE) and enhancing environmental quality. Mechanism analysis reveals that SCC achieves these effects through scale effects, structural effects, and technological effects. City heterogeneity analysis shows that provincial capital cities exhibit a stronger suppression effect on pollution emissions compared to non-provincial capital cities. Moreover, cities with lower levels of education attainment demonstrate a stronger ability to curb pollution emissions, while larger cities exhibit a more pronounced impact on mitigating pollution emissions. The marginal contributions of this study mainly consist of three aspects: Firstly, it enriches the literature on environmental impact factors by assessing, for the first time, the influence of SCC on PE. Secondly, a comprehensive approach is employed, integrating VSD, EFD, SO_2_ data, and economic and pollution data at the city level. Time-Varying DID is used to evaluate the policy effects of SCC. Finally, the study analyzes the impact mechanisms of SCC policy on environmental emissions from various perspectives.

## Introduction

Since the initiation of the reform and opening-up policy, China has experienced rapid economic growth. However, this economic development has come at the expense of the environment, as factors such as urban population growth, industrial expansion, transportation, and commodity circulation have led to substantial pollution emissions^1^. The conventional urban development model has resulted in adverse climate and environmental conditions^2^. The increasing volume of pollution emissions has not only given rise to a series of severe environmental issues but has also posed a significant threat to public health^3^. According to estimates from the Environmental Protection Agency, the World Bank, and the Chinese Academy of Sciences, China incurs economic losses equivalent to 10% of its gross domestic product annually due to environmental problems^4^. In response to urgent issues like environmental pollution, the United Nations has implemented the “2030 Agenda for Sustainable Development”^5^. However, despite the implementation of a series of policy measures, there remains a gap between pollution emissions and the targeted goals. Therefore, in-depth research on reducing pollution emissions is of crucial theoretical value and policy guidance significance.

Reducing pollutant emissions and protecting the environment is an urgent necessity for achieving sustainable economic development. Scholars have approached the study of factors influencing pollutant emissions from various perspectives, aiming to mitigate environmental pollution. For instance, research indicates that factors such as energy innovation^6^, environmental regulation^7,8^ , foreign direct investment (FDI)^9^, financial development^10–12^, international trade^13,14^, trading policies^15^, economic growth^16^, energy transition^9^, seasonal variations^17^, and urban form^18^ can all impact pollutant emissions. Pollutants settling on road surfaces, mixing with road dust, can have adverse long-term effects on ecosystems and human health^19^. However, there is limited research on the impact of SCC pilot projects on pollutant emissions.

China's urban development process reveals that the majority of pollutant emissions are concentrated in urban areas, raising the question of whether traditional urban development can escape environmental pollution. Can a transformation in the urban development model, promoting technological innovation, break the convention of high pollutant emissions in cities? Therefore, there is a need to explore a new urban development model. The emergence of SCC as a new urban development model is evident, with the Chinese government initiating SCC in 2012, initiating a reform of the traditional urban development model^3^. SCC represents a new form of urban evolution, essentially constituting a significant innovation based on advances in social technology and organizational change, which drives cities to ascend to higher forms^20^. Chu et al.^21^ argue that SCC can enhance a city’s technological level and resource allocation efficiency, leading to improved Environmental Quality (EEQ). Guo et al.^22^ find that SCC can accelerate a city's digitization and intelligence transformation. As a development strategy for cities in the new era, SCC not only strengthens the agglomeration effects of expanding urban scale but also mitigates the crowding effects resulting from excessive scale^3^. Therefore, the key to addressing environmental pollution issues in the urban development process lies not in whether cities are too large or too small but in whether there is a transformation in urban governance models and an enhancement in technological progress.

Simultaneously, SCC as an exogenous shock, provides an excellent quasi-natural experiment for investigating its impact on PE. Specifically, SCC, implemented as a novel urban development model starting in 2012, represents an innovation in traditional urban development^3^. According to data from the National Bureau of Statistics, by the end of 2023, the number of Smart City pilot projects in China has exceeded a hundred. As a strategic approach for urban development in the new era, SCC leverages information technology, digital technology, and other innovations to transform urban governance models, enhance industrial agglomeration effects, and address urban issues through technological innovation, thereby contributing to both urban economic development and pollution reduction^23^. Therefore, under the overarching goal of comprehensively achieving a beautiful China in the context of socialist modernization, it is worthwhile to explore the impact of SCC on environmental protection. Meanwhile, considering the different batches of Smart City pilot projects in various cities, studying the effects of SCC on PE in these diverse urban settings becomes feasible. By employing the Time-Varying DID method for investigation, this approach allows for a comprehensive verification of the impacts of policies over multiple periods.

Existing research has some limitations. The relevant studies on the impact of SCC on urban PE have not received sufficient attention from the academic community and policymakers. Until now, it remains unclear whether SCC can reduce environmental pollution emissions such as VSD, SO_2_, and EFD in cities. It is also unknown whether the PE of different cities will decrease due to SCC. In terms of theoretical and practical value, more efforts are needed to explore the mutual relationship between SCC and PE, as well as to investigate the underlying mechanisms of their influence.

Therefore, against the backdrop of deepening urbanization, this paper investigates the impact of the new urban development model on reducing PE. In comparison to existing research, this paper expands primarily in three areas: First, most existing literature predominantly focuses on the environmental costs associated with urban development. In contrast, this paper, for the first time, assesses the impact of SCC policies on PE, enriching the literature on factors influencing environmental protection and emission reduction. Second, this paper comprehensively utilizes data on wastewater discharge, particulate matter emissions, SO_2_ emissions, and economic and pollution data at the city level. Considering the first, second, and third batches of pilot cities, the paper employs a Time-Varying DID model to assess the policy effects of Smart Cities. Third, the paper analyzes the mechanism pathways through which SCC reduces PE, including scale effects, structural effects, technological effects, etc., providing a detailed examination of the impact mechanism pathways of SCC policies on PE.

The discussion in this paper follows the structure outlined below. Chapter 2 provides a review of the policy background and literature. Chapter 3 is part of the theoretical analysis and research hypothesis. Chapter 4 primarily introduces the model design, details the data used in this study, and outlines the research scope. Chapter 5 conducts empirical analysis of the data. Chapters 6 and 7 respectively delve into mechanism analysis and heterogeneity analysis. Chapter 8 presents the main conclusions and provides policy recommendations.

## Policy background and literature review

### Policy Background

Since the beginning of the twenty-first century, the development of the new generation of information and communication technologies has reached its peak. Countries around the world have successively embraced the idea of SCC. Currently, there are more than 1000 smart cities globally that have been initiated or are under development, and this number continues to grow.

In 2005, the European Union initiated the “i2010” program and followed up with the “SCC of Internet” program in 2006. During the same year, the United Kingdom launched the “Digital Britain” initiative, Ireland conducted the “T-CITY” experiment, and Germany introduced the “Smart Bay’’ project. Similarly, in Asia, SCC initiatives were put into practice. In 2004, South Korea implemented the “U-Korea” strategic plan, aiming to construct SCC that is environmentally friendly, digital, and smartly connected, leveraging information and communication technology. Singapore initiated the “Smart Nation 2015” plan in 2006, spanning a decade and focused on achieving a high degree of integration among the government, enterprises, individuals, and infrastructure to drive intelligent national development. In 2009, the Netherlands launched an SCC project based on Amsterdam and developed an open data sharing platform to promote digital applications, aiming to enhance urban management efficiency and residents' quality of life. During the same year, Japan introduced the “I-Japan” program to achieve smart management and operation of various public sectors. In 2009, the city of Dibikey conducted an SCC experiment, digitizing and connecting city resources, and providing intelligent responses to resident demands by analyzing and integrating large data information.

The Smart City trend has not bypassed China, where the government has actively embraced it. In 2013, they successively established three batches of SCC pilot city lists. In summary, SCC is expected to have a significant impact on various aspects of China.

### Literature review

Since 2008, when IBM introduced the concept of SCC, scholars have been keenly interested in its effectiveness in reducing environmental pollution^24,25^. Some researchers propose that with the development of cities and the advancement of new technologies like the digital economy, there is a significant potential to reduce pollutant emissions^26^. However, due to differences in the basic conditions and technological levels among countries, scholars have not reached a consensus on the pollution reduction and environmental protection effects of SCC^27^. On one hand, some scholars argue that SCC may increase pollution emissions. For instance, based on data concerning urban areas and air pollutants in 86 major U.S. metropolitan regions, Bereitschaft and Debbage^18^ found that urbanization exacerbates air pollution, increasing the concentration and emissions of carbon dioxide. Fang et al.^28^ suggest that urban development may intensify pollution emissions through the promotion of industrial scale and agglomeration, and investments in human capital and foreign direct investment do not necessarily reduce pollution emissions but rather enhance the effects of green growth. On the other hand, some scholars believe that SCC can lead to a reduction in pollution emissions. For example, Capodaglio and Olsson^29^ argue that SCC can bring about new technologies, allowing for more effective energy savings and energy recovery from wastewater treatment processes, thereby achieving energy recovery and pollution reduction. Zhao et al.^30^ propose that the development of SCC can significantly curb pollutant emissions through the implementation of intelligent transportation systems. Gao Da et al.^31^ contend that enhancing market competition and reducing overinvestment can provide enterprises with the impetus for technological innovation, leading to a reduction in environmental pollution.

At the same time, scholars have gradually explored the pollution reduction mechanisms of SCC. Chen^32^ discovered that the inhibitory effect of SCC on pollutant emissions is achieved through optimizing energy structure and advancing treatment technologies, rather than reducing energy consumption. Hu et al.^33^ propose that SCC can lower pollution levels in regions by enhancing the city’s level of informatization. Su et al.^34^ point out that SCC indirectly influences environmental pollution levels by improving industrial structural upgrades and resource allocation efficiency. However, these studies overlook the indirect effects of the scale, structural, and technological impacts generated by SCC on PE.

In addition, most scholars utilize the traditional DID method to investigate the economic impact of SCC. For example, Chu et al.^35^ employed the DID approach to study the influence of SCC on China's ecological environmental quality from 2005 to 2017, The results indicated that the implementation of SCC in pilot areas effectively reduced local pollutant emissions. Zhang and Wu^36^ applied the DID method to assess the impact of SCC pilot policies on the emission of haze pollutants in urban air. They found a significant reduction in haze pollution emissions as a result of SCC pilot implementation. Shen et al.^37^ used the DID model to explore the pathways through which SCC reduces environmental pollution. However, previous literature primarily focused on the first batch of SCC pilot cities and did not consider the three successive batches of pilot cities in the model. Since SCC was gradually implemented in these three batches of pilot cities, the traditional DID method has relative limitations in evaluating policy effects^38^. To address this limitation, scholars widely employ the Time-Varying DID model to investigate the impact of multiple policies on SCC in reducing PE.

In summary, previous literature has laid a certain foundation for the study of the economic effects of SCC, but there are some limitations: Firstly, existing studies on SCC’s impact on pollutant emissions mostly focus on air pollutants such as haze pollution and carbon emissions. Few scholars have investigated the effects of SCC on the emissions of pollutants like VSD, SO_2_, and EFD. Secondly, most research on SCC pilot programs in China has concentrated on the initial batch of 32 cities in 2013, neglecting the consideration of the second and third batches of pilot cities. Consequently, the traditional single-period DID approach may lead to inaccurate estimates. Thirdly, prior studies have not conducted a comprehensive analysis of the impact mechanism of SCC on PE, overlooking the mediating effects of scale, structure, and technological factors.

## Theoretical analysis and research hypothesis

SCC is a significant indicator of a country transitioning to the "innovation-driven" phase. The scale effects, structural effects, and technological effects generated by SCC, driven by innovation, are part of the innovation-driven process. These three effects collectively result in a continuous reduction in PE^35^.

### SCC can reduce PE

SCC has the potential to address environmental issues resulting from improper waste management by improving public health, safeguarding aquatic ecosystems, and reducing air pollution 38. Chandiramani^39^ conducted research on India's “Smart Cities Mission” initiated in 2015 and found that SCC-related projects with environmental relevance have been launched and are in progress, but they have yet to bring about significant environmental changes. Chen^40^, using nitrogen dioxide (NO_2_) as an indicator of air pollution, studied the impact of SCC on PE of air and concluded that SSC could potentially be the optimal path for developing countries to achieve the EKC (Environmental Kuznets Curve), balancing environmental pollution and economic development. Smart cities rely on networks and serve as new sources of real-time information. They use various devices to address pressing urban challenges such as resource shortages, environmental pollution, traffic congestion, and safety concerns^41^. Based on this, the hypothesis is proposed:

#### Hypothesis 1

SCC can directly reduce urban PE.

### SCC can reduce PE by scale effect

The scale effects of SCC primarily manifest in two aspects. 1. The concentration of production factors brought about by SCC can lead to the release of labor^42^, lower knowledge spillover costs, increase productivity, and reduce pollution emissions, thereby reducing PE^43^. Population agglomeration can not only reduce PE through green transportation but achieve pollution reduction by lowering the prices of intermediate inputs through the release of labor^44^, shared intermediate inputs, and knowledge spillover, as well as by enhancing productivity and pollution reduction technologies. 2. While improving production efficiency, SCC can trigger an expansion of production scale and an increase in production capacity, leading to the transformation of industries into intelligent ones. This transformation alters the traditional high-pollution industrial production scale and methods, effectively reducing emissions of pollutants. Based on this, the paper proposes:

#### Hypothesis 2

SCC exhibits a scale effect and will reduce PE through this scale effect.

### SCC can reduce PE by structural effect

The structural effect of SCC manifest in two main aspects:1. Utilization of Smart Elements and Industrial Structure Upgrading: In SCC, a significant utilization of smart elements leads to the industrial structure upgrading. This involves the elimination of traditional elements used by polluting industries and companies^45^. By altering and optimizing the composition of these elements and improving the production structure, SCC contributes to a reduction in PE^46^.2. Acceleration of Industrial Agglomeration and Regional Industrial Structure Upgrading: SCC accelerates the agglomeration of industries within urban areas and expedites the upgrading of regional industrial structures. It reduces pollution emissions and improves environmental quality due to its high technological content in industries^47^. Structural effects can improve the industrial structure, balance environmental pollution with economic development, ameliorate environmental pollution, and reduce PE. They contribute to environmental pollution improvement and PE reduction^48,49^. Building on this, the paper proposes:

#### Hypothesis 2

SCC possesses a structural effect and will reduce PE through this structural effect.

### SCC Can reduce PE by technological effect

The technological effects of SCC primarily manifest in two key aspects: 1. Advancement of Production, Energy Efficiency, and Environmental Technologies: Technological effects foster the progress of production technologies, energy-saving technologies, and environmental technologies, resulting in a reduction of PE^50^. Cui and Cao^51^ found that the impact of SCC on PM2.5 primarily stems from the technological effects generated through the promotion of innovation and distribution effects. SCC implements real-time monitoring through intelligent monitoring systems, enhancing companies' ability to monitor environmental pollution and access pollution data. This enables targeted control and management of various pollutants and pollution sources, promoting environmental protection^52^. 2. Promotion of Clean Energy Development: Smart city technological innovation increases the use of clean energy, and replaces polluting energy sources. This leads to improved energy efficiency, changes in energy consumption patterns, and reduced energy consumption, ultimately resulting in lower pollution emissions^53^. Technological effects enhance urban production efficiency, reduce energy usage, and lower PE, all while promoting economic growth^48^. Building on this, the paper proposes:

#### Hypothesis 4:

SCC exhibits a technological effect and will reduce PE through this technological effect.

**Figure 1:**
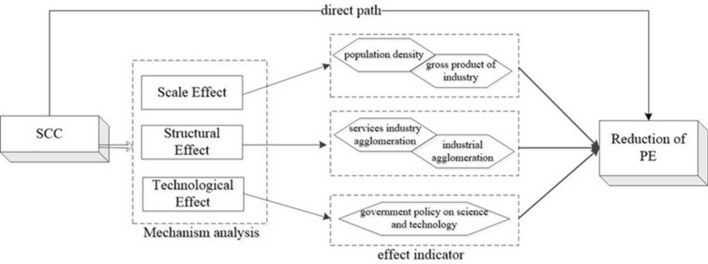
Mechanism Path Analysis.

After the theoretical analysis presented above, a schematic diagram of the mechanistic pathway (as in Fig. 1) is derived.

## Research design

### Model design

This paper employs three batches of Chinese SCC lists to construct a quasi-natural experiment. It utilizes a multi-time varying DID model to identify the policy impact of SCC on PE.

1 $$\begin{array}{c}PE\left\{\begin{array}{c}{VSD}_{it}\\ {{SO}_{2}}_{it}\\ {EFD}_{it}\end{array}={\alpha }_{0}+{\alpha }_{1}{SCCdid}_{it}+\sum_{i=2}^{n}{\alpha }_{i}{Control}_{it}+{\mu }_{i}+{\lambda }_{t}+{\varepsilon }_{it}\right. \end{array}$$

In the above equations: $i$ and $t$ are city and year, respectively. ${VSD}_{it}$,${{SO}_{2}}_{it}$ and ${EFD}_{it}$ represent PE indicators. ${SCCdid}_{it}$ represents the Smart City Construction (SCC) treatment variable. ${Control}_{it}$ stands for control variables. ${\mu }_{i}$ and ${\lambda }_{t}$ are fixed effects for individual cities and time periods. ${\varepsilon }_{it}$ represents the random disturbance term.

Considering the regional disparities in China's development, ensuring consistency among different samples may be challenging. Therefore, the PSM-DID method is employed to ensure the scientific rigor and accuracy of the analysis.

2 $$\begin{array}{c}PE\left\{\begin{array}{c}{{VSD}_{it}}^{PSM}\\ {{{SO}_{2}}_{it}}^{PSM}\\ {{EFD}_{it}}^{PSM}\end{array}\right.={\beta }_{0}+{\beta }_{1}{SCCdid}_{it}+\sum_{i=2}^{n}{\beta }_{i}{Control}_{it}+{\mu }_{i}+{\lambda }_{t}+{\varepsilon }_{it}\end{array}$$

The impact of SCC on PE falls within the realm of policy effect assessment, thus warranting an exploration of its mechanisms. The model is presented as follows:

The first-step:

3 $$\begin{array}{c}Effect\left\{\begin{array}{c}Scale{E}_{it}\\ {StrE}_{it}\\ {TechE}_{it}\end{array}\right.={b}_{0}+{b}_{1}{SCCdid}_{it}+\sum_{i=2}^{n}{b}_{i}{Control}_{it}+{\mu }_{i}+{\lambda }_{t}+{\varepsilon }_{it}\#\end{array}$$

The second-step:

4 $$\begin{array}{c}PE\left\{\begin{array}{c}{VSD}_{it}\\ {{SO}_{2}}_{it}\\ {EFD}_{it}\end{array}\right.={c}_{0}+{c}_{1}{SCCdid}_{it}+{c}_{2}Effect\left\{\begin{array}{c}Scale{E}_{it}\\ {StrE}_{it}\\ {TechE}_{it}\end{array}\right.+\sum_{i=3}^{n}{c}_{i}{Control}_{it}+{\mu }_{i}+{\lambda }_{t}+{\varepsilon }_{it}\end{array}$$

$Scale{E}_{it},{StrE}_{it}$ and ${TechE}_{it}$ represent the scale effect, structural effect, and technological effect, respectively.

### Variable selection

*Pollution emissions (PE)* Environmental pollution in cities is represented by the Volume of Sewage Discharge (VSD), Emissions of Fumes and Dust (EFD), and sulfur dioxide emissions (SO_2_).

*Smart city (*$SCCdid$*)* Smart Cities are represented as a binary variable. In accordance with the Time-Varying DID model, the variables are processed as follows:

1. *Treatment and control group binary variables (*$treat$*)* The treatment group represents the pilot cities and is assigned a value of 1, while the non-SCC cities are assigned to 0.
2. *Year binary variable (*$time$*)* Years before 2013 are assigned to 0, and else are assigned to 1.
3. *Time-varying DID variable (*$SCC{did}_{it}$*)* This is an interaction variable between time and year, denoted as ${time}_{it}*{treat}_{it}$.

Control variables:

1. *Economic development* City economic development is represented by variables such as regional GDP, per capita regional GDP, and urbanization levels.
2. *Industrial structure* The city’s GDP of secondary industry to the city's administrative area.
3. *Openness to foreign investment* The degree of openness to foreign investment is represented by the ratio of FDI to GDP.
4. *Green development* Urban greenery is represented by variables such as park and green space area.

Descriptive statistics are presented in Table 1.

**Table 1:** Descriptive statistics for variables.

Variables	Meanings of the variables	N	Mean	SD	Min	Max
*PE*						
$VSD$	Volume of sewage discharge	3990	5.429	5.805	0.004	70.962
$$ lnEFD$$	Emissions of fumes and dust	3990	9.566	1.225	0.693	15.458
$l nso2$	Sulfur dioxide emissions	3990	10.273	1.511	0.693	14.569
$SCCdid$	Smart city construction (SCC)	3990	0.209	0.406	0.000	1.000
$lngdp$	GDP	3990	6.809	0.960	3.718	9.782
$lnpergdp$	Per capita GDP	3990	9.552	1.338	5.480	12.513
$urban$	Urbanization level	3990	32.614	21.658	3.412	100.000
$fdigdp$	Openness	3990	0.017	0.020	0.002	0.376
$gardon$	Green space area	3990	6.469	0.914	2.773	9.693
$secagg$	Industrial structure	3990	0.083	0.132	0.000	1.695

The data is sourced from various references, including the "China City Statistical Yearbook," the National Bureau of Statistics, and government work reports. For missing values, a combination of moving average smoothing and interpolation methods has been used to fill in the gaps.

To avoid potential bias in the model estimation due to high intercorrelations between variables, this study conducted a multicollinearity test on the variables included in the model. The mean VIF is 4.50, which is significantly below 10.

### Data description

The study designates the Smart Cities established from 2013 onwards as the treatment group. This includes the first pilot (32 cities), the second pilot (37 cities), and the third pilot (24 cities). In total, the sample consists of 210 cities, with 93 Smart Cities are treatment group and 117 cities are control group (as shown in Table 2).

**Table 2:** List of SCC and non-SCC.

SCC	non-SCC
First Pilot: Shijiazhuang City, Handan City, Langfang City, Taiyuan City, Changzhi City, Wuhai City, Liaoyuan City, Wuxi City, Changzhou City, Zhenjiang City, Taizhou City, Wenzhou City, Jinhua City, Wuhu City, Huainan City, Tongling City, Nanping City, Pingxiang City, Dongying City, Weihai City, Dezhou City, Zhengzhou City, Hebi City, Luohe City, Wuhan City, Zhuzhou City, Changde City, Zhuhai City, Ya'an City, Liupanshui City, Xianyang City, Wuzhong City Second Pilot: Yangquan City, Jincheng City, Ordos City, Hulunbuir City, Yingkou City, Siping City, Qiqihar City, Mudanjiang City, Nantong City, Huaibei City, Huangshan City, Fuyang City, Putian City, Xinyu City, Yantai City, Xuchang City, Yichang City, Xiangyang City, Jingzhou City, Huanggang City, Xiantao City, Ezhou City, Suizhou City, Jiujiang City, Nanning City, Liuzhou City, Guilin City, Qinzhou City, Guigang City, Yulin City, Luzhou City, Mianyang City, Suining City, Guiyang City, Zunyi City, Baoji City, Weinan City, Yan'an City, Lanzhou City, Jinchang City, Baiyin City, Tianshui City, Zhangye City, Yinchuan City, Shizuishan City, Urumqi City Third Pilot: Tangshan City, Datong City, Xinzhou City, Hohhot City, Tonghua City, Jiamusi City, Chuzhou City, Suzhou City, Bozhou City, Quanzhou City, Yingtan City, Ji'an City, Kaifeng City, Nanyang City, Jingzhou City, Qinzhou City, Yulin City, Zigong City, Guang'an City, Yuxi City, Hanzhong City, Tianshui City, Zhangye City	Xingtai City, Baoding City, Zhangjiakou City, Chengde City, Cangzhou City, Hengshui City, Jinzhong City, Yuncheng City, Linfen City, Lvliang City, Chifeng City, Bayan Nur City, Ulanqab City, Fushun City, Benxi City, Dandong City, Jinzhou City, Fuxin City, Panjin City, Tieling City, Huludao City, Songyuan City, Baicheng City, Jixi City, Hegang City, Yichun City, Suihua City, Yangzhou City, Jiaxing City, Huzhou City, Shaoxing City, Quzhou City, Zhoushan City, Taizhou City, Lishui City, Ma'anshan City, Anqing City, Chizhou City, Xuancheng City, Xiamen City, Sanming City, Longyan City, Ningde City, Jingdezhen City, Jiujiang City, Ganzhou City, Yichun City, Zibo City, Tai'an City, Linyi City, Liaocheng City, Binzhou City, Heze City, Luoyang City, Pingdingshan City, Anyang City, Xinxiang City, Jiaozuo City, Puyang City, Sanmenxia City, Shangqiu City, Xinyang City, Zhoukou City, Zhumadian City, Shiyan City, Ezhou City, Jingmen City, Xiaogan City, Suizhou City, Hengyang City, Shaoyang City, Zhangjiajie City, Yiyang City, Huaihua City, Loudi City, Shaoguan City, Shantou City, Jiangmen City, Zhanjiang City, Maoming City, Meizhou City, Shanwei City, Yangjiang City, Qingyuan City, Chaozhou City, Yunfu City, Wuzhou City, Beihai City, Fangchenggang City, Baise City, Hezhou City, Laibin City, Chongzuo City, Haikou City, Sanya City, Zigong City, Deyang City, Guangyuan City, Neijiang City, Nanchong City, Meishan City, Dazhou City, Bazhong City, Ziyang City, Qujing City, Baoshan City, Zhaotong City, Xi'an City, Tongchuan City, Yulin City, Ankang City, Shangluo City, Wuwei City, Pingliang City, Jiuquan City, Qingyang City, Xining City

## Results

### Benchmark analysis

SCC, as a new generation of information and technology, imparts intelligence to objects, integrates urban governance systems and services, achieves precise management of various aspects of the city, and facilitates the efficient utilization of urban resources. Furthermore, SCC significantly drives the development of strategic emerging industries, including cloud computing, the convergence of three networks, new internet technologies, and new information technologies, thereby effectively influencing China's structural adjustments and the transformation of its economic development mode. Therefore, this paper employs the Time-Varying DID method to investigate the impact of SCC on PE.

In Table 3, models (1)–(3) represent the baseline regression models for the impact of SCC on VSD, EFD, and lnSO2. Indeed, SCC does have a negative effect on VSD, EFD, and lnSO2. Specifically, SCC significantly reduces VSD by approximately 102.5%, while it also significantly decreases lnSO2 by about 65.4% and EFD by 18.8%. This suggests that SCC significantly reduces PE.

**Table 3:** Difference-in-difference model (DID) regression results.

	(1)	(2)	(3)	(4)	(5)	(6)
	$VSD$	$lnSO2$	$lnEFD$	$VSD$	$lnSO2$	$lnEFD$
$did$	− 1.025***	− 0.654***	− 0.188***	− 0.953***	− 0.113*	− 0.180***
	(− 5.49)	(− 11.47)	(− 4.25)	(− 5.06)	(− 1.71)	(− 4.14)
$lngdp$				1.756*	0.549	1.561***
				(2.34)	(1.13)	(7.86)
$lnpergdp$				− 1.267	− 0.845	− 1.373***
				(− 1.60)	(− 1.68)	(− 6.97)
$urban$				− 0.0281*	− 0.019***	0.002
				(− 2.42)	(− 4.32)	(0.61)
$fdigdp$				− 10.17**	− 4.037**	− 6.255***
				(− 3.14)	(− 2.90)	(− 6.62)
$gardon$				0.552*	− 0.123*	0.007
				(2.31)	(− 2.12)	(0.16)
$secagg$				− 3.018	− 1.751***	− 1.132***
				(− 1.30)	(− 3.35)	(− 4.54)
$Year FE$	$TRUE$	$TRUE$	$TRUE$	$TRUE$	$TRUE$	$TRUE$
$City FE$	$TRUE$	$TRUE$	$TRUE$	$TRUE$	$TRUE$	$TRUE$
$N$	3990	3989	3990	3989	3988	3989
${R}^{2}$	0.7372	0.7820	0.6882	0.7401	0.7842	0.6984

The values in parentheses refer to the t-values of the regression coefficients; ***, **, and * refer to the significance levels of 1%, 5%, and10%, respectively.

Models (4)–(6) represent the regressions after including control variables in Models (1)–(3). It's also found that SCC can suppress PE. However, the introduction of control variables leads to changes in the magnitude of SCC’s impact on VSD, EFD, and lnSO2. Specifically, SCC's effect on VSD decreases from 102.5 to 95.3%, its effect on lnSO2 decreases from 65.4 to 11.3%, and its effect on EFD increases from 18.8 to 18.0%. However, after including control variables, SCC's significant inhibitory effect on sulfur dioxide emissions is significantly reduced. In Table 2, the role of SCC in curbing urban environmental pollution is achieved through various pathways. It can be argued that only the policy impact of SCC is evident, with a substantial and more significant effect. However, when control variables are included, the degree of policy impact decreases, and its significance slightly diminishes. This suggests that the effect of SCC in suppressing PE is manifested through the pathways reflected by the control variables, and the policy's own impact decreases.

### Robustness check

#### Parallel trend test

The estimation of Time-Varying DID is effective only if the parallel trends assumption holds. Therefore, it's essential to assess the consistency of time trends before comparing SCC before and after. In this paper, using Time-Varying DID, the main focus is on whether the estimated parameters of SCC and non-SCC exhibit consistent parallel trends before the introduction of SCC.

**Figure 2:**
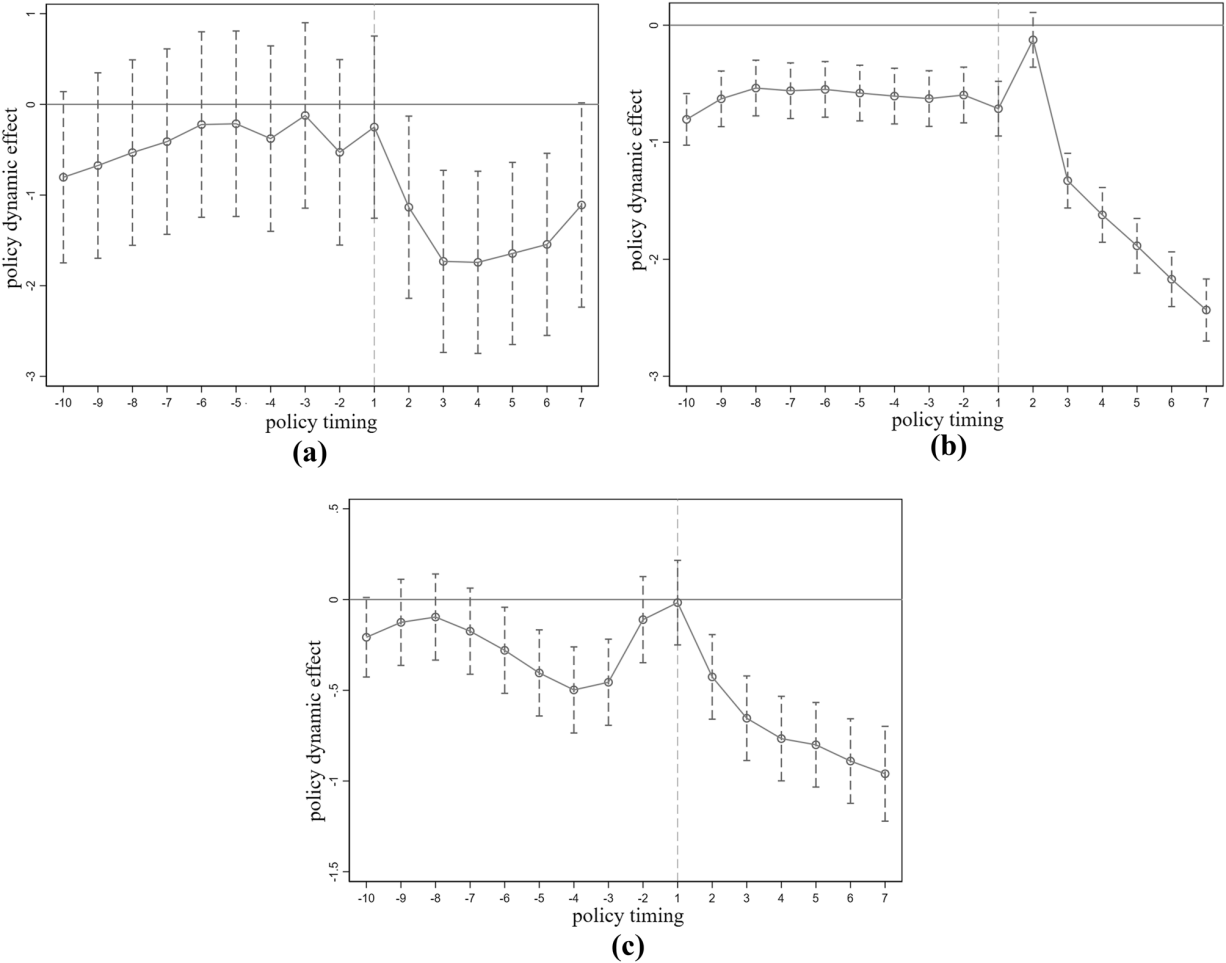
The parallel trend test results of VSD, SO_2_ and EFD.

Figures 2a–c represent the parallel trends test results for VSD, SO_2_, and EFD. The estimated coefficient for SCC is not statistically significant, but after the implementation of SCC, VSD, SO_2_, and EFD all show significant decreases. This indicates that before the implementation of SCC, the time trends remained consistent, thus satisfying the parallel trends assumption for DID. At the same time, after SCC, the policy estimate coefficients continue to decrease. This suggests that SCC has had a significant policy impact on VSD, SO_2_, and EFD, demonstrating a noticeable suppressive effect on PE.

#### Test of placebo

1. *Time-placebo tests* Time-placebo tests are employed to ensure that the differences in the impact of SCC and non-SCC on PE are due to temporal changes. In this paper, SCC is advanced in time by 2 years, 3 years, 4 years, and 5 years, constructing false policy times, respectively, creating false policy times represented by ${SCCdid}^{false2}$, $SCC{did}^{false3}$,$SCC{did}^{false4}$,$SCC{did}^{false5}$. The regression analyses were then performed on EFD, VSD, and lnSO2 for each of these false policy times.
  The results show that (in Table 4), under the premise of controlling for other influencing factors, the regression coefficient estimates for VSD, EFD, and lnSO2 did not pass the tests, regardless of the specific false policy time used. This indicates that the time trends of SCC do not exhibit systematic differences and reaffirms that SCC effectively suppresses PE.

**Table 4:** The time placebo test results for VSD, SO_2_, and EFD with lags of stages 2, 3, 4, and 5.

$lnEFD$	(1)	(2)	(3)	(4)
	${SCCdid}^{false2}$	${SCCdid}^{false3}$	${SCCdid}^{false4}$	${SCCdid}^{false5}$
Panel A: EFD				
$SCCdid$	− 0.192	− 0.204	− 0.197	− 0.196
	(− 0.01)	(− 0.24)	(− 0.25)	(− 0.63)
${R}^{2}$	0.6985	0.6987	0.6985	0.6983
Panel B: VSD				
$SCCdid$	− 0.0494	− 0.0501	− 0.0488	− 0.0567
	(− 0.40)	(− 0.39)	(− 0.38)	(− 0.41)
${R}^{2}$	0.7842	0.7842	0.7842	0.7842
Panel C: SO_2_				
$SCCdid$	− 1.044	− 1.003	− 1.065	− 1.096
	(− 1.05)	(− 1.03)	(− 1.09)	(− 1.12)
$Control$	TRUE	TRUE	TRUE	TRUE
$Year FE$	TRUE	TRUE	TRUE	TRUE
$City FE$	TRUE	TRUE	TRUE	TRUE
$N$	3990	3990	3990	3990
${R}^{2}$	0.7405	0.7403	0.7404	0.7403

**Figure 3:**
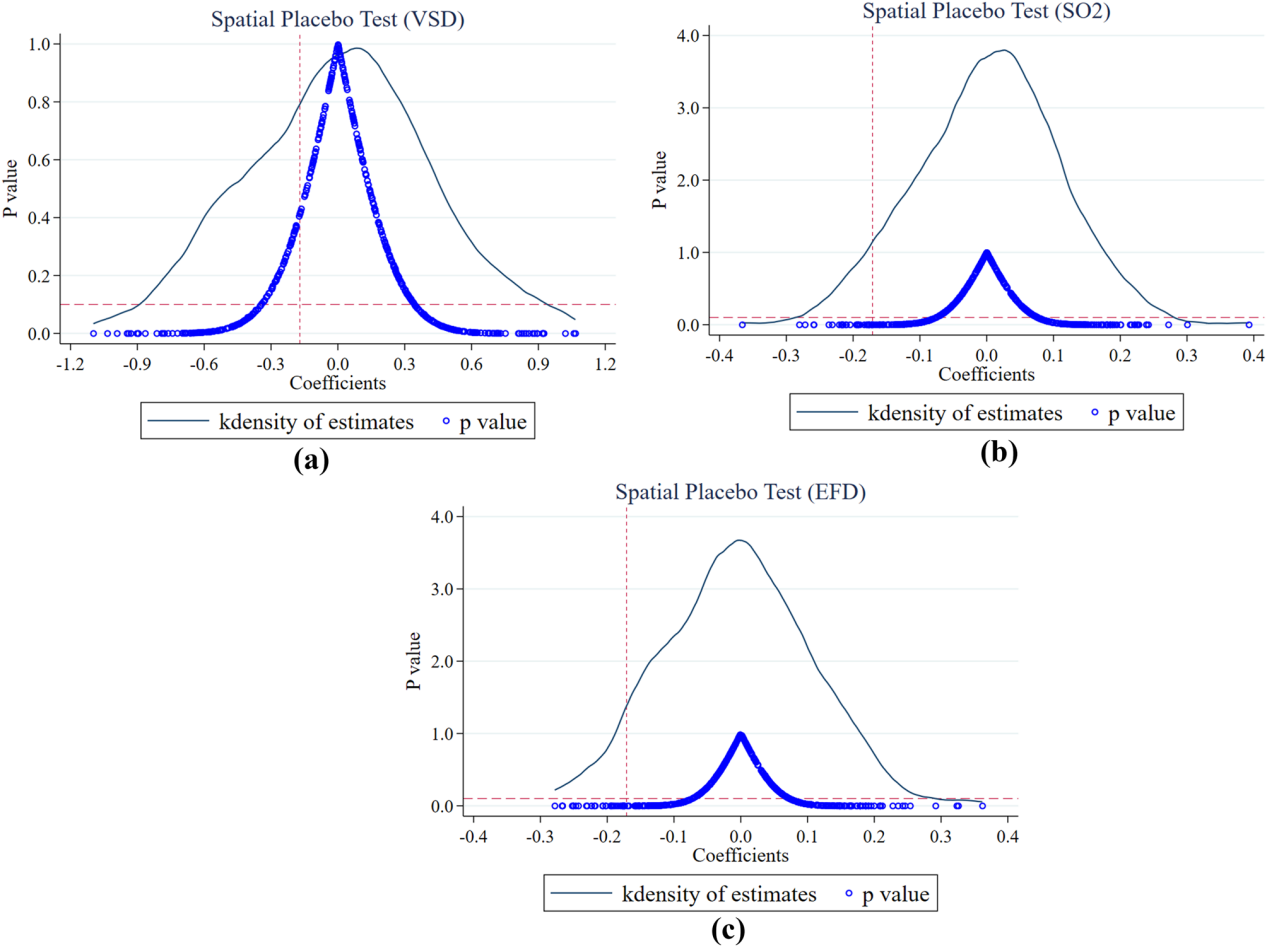
The spatial placebo test results of VSD, SO_2_ and EFD.

All coefficients in the table are statistically insignificant. It is preferable for the coefficient of the time placebo DID variable to be non-significant.
2. *Spatial placebo test* Considering the possibility of unobserved variables affecting the baseline regression results and causing biased estimated coefficients, this study conducts a placebo test. In this test, a random set of 100 cities from the sample is chosen as a fictitious SCC, while the remaining cities serve as non-SCC. The coefficients of SCC's impact on PE under the placebo treatment are estimated. We repeated this process 500 times, obtaining 500 false estimation coefficients and* p* values, then plotted the kernel density distribution of these 500 coefficients^54^. Figures 3a–c represent the city placebo test results for VSD, SO_2_, and EFD. Figure 3 illustrates that the estimates from random assignment are concentrated around 0, indicating no substantial impact on the pseudo-treated sample^55^. This observation suggests that the observed effects of SCC on PE are not spurious. Therefore, the results of the placebo test enhance our confidence in the regression results, further confirming the significant impact of SCC on PE.

#### PSM-DID test

To mitigate potential systematic bias in the comparative trends between SCC and non-SCC address endogeneity issues resulting from self-selection, this study uses the PSM-DID method for further examination. Considering the disparities in variables such as $gardon$, $secagg$, $lngdp$, $urban$, $fdigdp$, $lnpergdp$ among others, the distinguishing characteristics between the cities mentioned earlier are utilized as matching variables. The non-SCC cities are matched to the SCC cities that are more similar, and a analysis is conducted with the matched control group. If all variables exhibit significantly reduced standardized differences after matching, it indicates that there are no significant differences after matching, and a DID analysis can be carried out. Matching is performed on these variables using kernel density matching, and matching scores are calculated. After matching, both sets of data exhibit high consistency and significantly reduced standardized differences, which conforms to the common trends assumption. This provides a basis for conducting the analysis.

Table 5 presents the results of the PSM-DID analysis. When controlling for time, cities, and including control variables, the coefficients for SCC on the three PE indicators remain negative. This helps eliminate the potential bias introduced by sample selection and confirms the accuracy of the baseline regression results, indicating that SCC has a mitigating effect on PE.

**Table 5:** PSM-DID regression results of VSD, SO_2_ and EFD.

	(1)	(2)	(3)
	$VSD$	$SO2$	$EFD$
$SCCdid$	− 1.0737***	− 0.1629**	− 0.2737**
	(− 3.7708)	(− 2.6164)	(− 3.0112)
$lngdp$	1.2971***	− 2.9346***	− 2.0700***
	(5.9570)	(− 14.1920)	(− 6.3492)
$lnpergdp$	0.9974***	2.4344***	1.7632***
	(6.2393)	(11.3532)	(4.9973)
$urban$	− 0.0885***	− 0.0133***	− 0.0261***
	(− 20.1885)	(− 4.7729)	(− 5.7743)
$fdigdp$	− 1.0591	5.1318***	− 2.6654**
	(− 0.3974)	(6.0858)	(− 2.3965)
$gardon$	1.3296***	0.3009***	− 0.0318
	(7.1662)	(6.9948)	(− 0.6015)
$secagg$	17.3918***	− 1.1458***	− 0.5113***
	(50.7185)	(− 9.3107)	(− 3.0904)
$N$	4080	4064	4064
${R}^{2}$	0.7628	0.9077	0.8700

The values in parentheses refer to the t-values of the regression coefficients; ***, **, and * refer to the significance levels of 1%, 5%, and10%, respectively.

#### Other robustness tests

1. Sample data selection
  Sample data screening is performed to avoid the influence of outliers on the regression and to obtain general conclusions. Therefore, tail truncation at both the 1% and 5% levels is applied to the research sample, followed by a new round of regression estimation (Table 6). The estimation results indicate that, after excluding extreme values of variables, the coefficients of the DID remain negative. Specifically, VSD and lnEFD remain highly significant, but the 1% trimmed regression results for lnSO_2_ are not significant, while the 5% trimmed regression results are significant at the 10% level. This is because, after the 1% trimming process, the number of outliers is reduced to some extent, but scatter plots reveal that outliers still exist, leading to a decrease in the interpretability of the data. However, at the 5% trimming level, the scatter plot shows that the number of outliers decreases further, restoring the interpretability of SCC's impact on PE. Overall, this conclusion is consistent with the baseline estimation results.

**Table 6:** Regression results for VSD, SO_2_, and EFD shrinkage of 1% and 5% respectively.

	(1)	(2)	(3)	(4)	(5)	(6)
	$VSDw1$	$VSDw5$	$lnso2w1$	$lnso2w5$	$lnEFDw1$	$lnEFDw5$
$SCCdid$	− 0.939***	− 0.543***	− 0.0612	− 0.0829**	− 0.171***	− 0.148***
	(− 5.67)	(− 4.20)	(− 1.41)	(− 2.15)	(− 4.21)	(− 4.11)
$Control$	$TRUE$	$TRUE$	$TRUE$	$TRUE$	$TRUE$	$TRUE$
$Year FE$	$TRUE$	$TRUE$	$TRUE$	$TRUE$	$TRUE$	$TRUE$
$City FE$	$TRUE$	$TRUE$	$TRUE$	$TRUE$	$TRUE$	$TRUE$
$N$	3989	3989	3988	3988	3989	3989
${R}^{2}$	0.7918	0.8049	0. 7998	0.8083	0.7144	0.7070

The values in parentheses refer to the t-values of the regression coefficients; ***, **, and * refer to the significance levels of 1%, 5%, and10%, respectively.
2. Exclusion of Special Year Effects
  In 2003 and 2020, global pandemics, namely the SARS epidemic and the COVID-19 pandemic, swept across the world, impacting policies in China and influencing social development. Recognizing the influence of these special years on SCC, this study excludes the data for these two years to assess the robustness of the model (see Table 7). The results indicate that even after excluding data from 2003 and 2020, SCC continues to have a negative impact on VSD, lnSO2, and EFD. This suggests that the model is relatively robust, with the estimation process being less affected by the policy impacts of these special years.

**Table 7:** Exclusion of special year regression results of VSD, SO_2_ and EFD.

	(1)	(2)	(3)
	$VSD$	$lnso2$	$lnEFD$
$SCCdid$	− 0.952***	− 0.348***	− 0.155**
	(− 4.62)	(− 5.34)	(− 3.22)
$Control$	$TRUE$	$TRUE$	$TRUE$
$Year FE$	$TRUE$	$TRUE$	$TRUE$
$City FE$	$TRUE$	$TRUE$	$TRUE$
$N$	3360	3359	3360
${R}^{2}$	0.7461	0.7826	0.6940

The values in parentheses refer to the t-values of the regression coefficients; ***, **, and * refer to the significance levels of 1%, 5%, and10%, respectively.
3. Changing variables
  To determine whether the impact of SCC on PE is influenced by factors outside the model, we added three variables, Comprehensive utilization rate of solid waste ($Cursw$), Non-hazardous waste disposal rate ($Nwdr$), and Area covered by greenery ($mm$), to the control variables for regression estimation (see Table 8). The results show that, even after adding new control variables, the impact of $SCC$ on $VSD$ and $lnEFD$ remains unchanged, and the direction of the coefficients remains the same. This indicates that the model is relatively robust and has incorporated other significant influencing factors outside the core explanatory variables. The model estimation results are robust. However, the impact of SCC on SO_2_ shifts from significant to non-significant. This change occurs because the SCC-DID coefficient, now accounting for the influence after controlling for additional variables, transitions from significant to non-significant. The reason behind this lies in the introduction of additional control variables, among which there are competing variables with the core explanatory variable, such as waste utilization rate. Moreover, these competing variables exhibit a higher impact than the core explanatory variable. Therefore, it is worthwhile to further investigate their moderating effects in the relationship between SCC and SO_2_.

**Table 8:** Regression results after changing VSD, SO_2_ and EFD measures.

	(1)	(2)	(3)
	$VSD$	$lnso2$	$lnEFD$
$SCCdid$	− 0.958***	− 0.058	− 0.183***
	(− 5.112)	(− 1.210)	(− 4.126)
$mm$	− 0.001	− 0.001	− 0.001
	(− 0.134)	(− 0.211)	(− 1.087)
$Cursw$	0.002	0.002***	0.001
	(0.821)	(3.165)	(0.481)
$Nwdr$	− 0.001***	− 0.001	− 0.001
	(− 2.678)	(− 0.726)	(− 1.606)
$cons$	− 2.630	− 0.701	− 1.169***
	(− 1.046)	(− 1.479)	(− 4.426)
$Control$	$TRUE$	$TRUE$	$TRUE$
$Year FE$	$TRUE$	$TRUE$	$TRUE$
$City FE$	$TRUE$	$TRUE$	$TRUE$
$N$	3989	3988	3989
${R}^{2}$	0.7232	0.7705	0.6795

Note: The values in parentheses refer to the t-values of the regression coefficients; ***, **, and * refer to the significance levels of 1%, 5%, and10%, respectively.
4. Lagged period
  To eliminate the interference of policy lag on the model estimation error, a lagged period model is employed, with explanatory variables lagged by one and two periods, followed by regression estimation (see Table 9). The results demonstrate that whether lagged by one or two periods, the DID estimated coefficients remain consistent with the original baseline model and are consistently statistically significant in the negative direction. Therefore, the conclusions of this study are robust.

**Table 9:** Regression results with lagging of VSD, SO_2_ and EFD.

	(1)	(2)	(3)
	$VSD$	$lnso2$	$lnEFD$
Panel A: $L. SCCdid$			
$L. SCCdid$	− 0.775***	− 0.268***	− 0.152***
	(− 4.04)	(− 3.82)	(− 3.34)
${R}^{2}$	0.7417	0.7859	0.6997
Panel B: $L2. SCCdid$			
$L2. SCCdid$	− 0.605**	− 0.660***	− 0.126**
	(− 3.11)	(− 9.02)	(− 2.62)
$Control$	$TRUE$	$TRUE$	$TRUE$
$Year FE$	$TRUE$	$TRUE$	$TRUE$
$City FE$	$TRUE$	$TRUE$	$TRUE$
$N$	3570	3569	3570
${R}^{2}$	0.7468	0.7890	0.7032

The values in parentheses refer to the t-values of the regression coefficients; ***, **, and * refer to the significance levels of 1%, 5%, and10%, respectively.

## Mechanisms

The above analysis confirms the impact of SCC on PE. The baseline estimates show that policy implementation contributes to mitigating PE, thus promoting green economic development in China. However, further research is needed to understand the mechanisms through which SCC affects sustainable economic development. This paper delves into the transmission mechanisms of how SCC impacts PE from three angles: scale effects represented by population density and industrial output, structural effects measured by the agglomeration of the secondary and tertiary industry, and technological effects represented by government technology policies.

1. Scale effect
  SCC can influence PE through population density ($lnpeo$). SCC may attract talent and high-tech companies through preferential policies, which can affect PE by influencing population agglomeration. A lower population density implies longer vehicle travel distances and more fuel consumption, which can increase PE^56^. Additionally, the industrial output reflects the level of industrial development, and SCC might integrate information and communication technology and the Cyber-Physical System (CPS) into industrial development. By attracting businesses to establish smart factories and applying intelligent production technology in production logistics management, human–machine interaction, and 3D technology, SCC can transform industries into intelligent production, change traditional high-pollution industrial production scales and methods, and effectively reduce pollutant emissions. This paper examines the relationship between SCC and $lnpeo$ and PE and $gy1$ and PE to test this mechanism (in Table 10). Population density is defined as the total population of an area divided by its land area. As per Table 10, SCC significantly increases population density, indicating that smart cities do indeed exhibit scale effects. The SCC has a positive impact on urban development, aligning with the theoretical analysis of scale effects. The results in Panel A columns (2–4) show that after incorporating population density and DID into the regression equation simultaneously, population density increases VSD and lnSO2, but its effect on EFD is not significant. This suggests that SCC cannot mitigate PE through population density. Conversely, as seen in Panel B, the industrial production scale effectively reduces environmental pollution. This indicates that SCC can alleviate urban environmental pollution through the scale effect of industrial production.

**Table 10:** Regression results of the mechanism test for the scale effect.

	(1)	(2)	(3)	(4)
	$lnpeo/gy1$	$VSD$	$lnEFD$	$lnso2$
Panel A:$lnpeo$				
$lnpeo$		0.751**	0.119	0.230***
		(2.76)	(1.91)	(3.56)
$SCCdid$	0.0261*	− 1.044***	− 0.191***	− 0.0932*
	(2.25)	(− 5.39)	(− 4.29)	(− 2.03)
$cons$	12.59***	− 3.811	8.101***	7.401***
	(3325.90)	(− 1.11)	(10.29)	(9.12)
${R}^{2}$	0.9270	0.7378	0.6885	0.7827
Panel B:$gy1$				
$gy1$		− 0.534***	− 0.0579***	− 0.130***
		(− 16.55)	(− 7.09)	(− 11.38)
$SCCdid$	0.375***	− 1.539***	− 0.193***	− 0.0642
	(4.22)	(− 8.72)	(− 4.32)	(− 1.03)
$Control$	$TRUE$	$TRUE$	$TRUE$	$TRUE$
$YearFE$	$TRUE$	$TRUE$	$TRUE$	$TRUE$
$City FE$	$TRUE$	$TRUE$	$TRUE$	$TRUE$
$N$	3989	3989	3989	3988
${R}^{2}$	0.8283	0.7407	0.6270	0.5184

The values in parentheses refer to the t-values of the regression coefficients; ***, **, and * refer to the significance levels of 1%, 5%, and10%, respectively.
2. Structural effect
  SCC's impact on PE can be represented using urban industrial structure as a proxy for structural effects^57^. SCC policies promote the transformation of the industrial structure toward environmentally friendly sectors like the service industry, driving the transition from resource-intensive industries to knowledge and technology-intensive sectors. To examine this mechanism, this study tests the effects of SCC on the services industry agglomeration (AGG3) and industrial agglomeration (AGG2). The estimation results are reported in Table 11. In Table 11, the coefficients of SCC are both significant, and their directions are opposite. This indicates that SCC promotes the agglomeration of the service sector while inhibiting the agglomeration of the secondary sector. Furthermore, the extent of inhibition is stronger compared to the promoting effect, which corroborates the earlier point regarding industrial structural transformation. The inhibitory effect on industrial clustering is stronger than the promotion effect on the service industry, supporting the earlier notion of industrial structural transformation. Panel A include DID and AGG3 in the model, showing that SCC significantly mitigates PE through AGG3, which itself significantly reduces urban environmental pollution. In Panel B, both DID and AGG2 are incorporated into the model. The results reveal that while SCC itself suppresses industrial clustering, it also has a PE-reducing effect. However, AGG2 significantly increases wastewater, sulfur dioxide, and dust emissions, exacerbating urban environmental pollution. Taking both into account, SCC effectively adjusts the industrial structure, promotes the shift of industries towards the service sector, suppresses industrial agglomeration, and, through structural effects, inhibits PE.

**Table 11:** Regression results of the mechanism test for the structural effect.

	(1)	(2)	(3)	(4)
	$AGG3$/$AGG2$	$VSD$	$lnEFD$	$lnso2$
Panel A:$AGG3$				
$AGG3$		− 3.290***	− 1.560***	− 3.552***
		(− 5.94)	(− 11.47)	(− 18.79)
$SCCdid$	0.0217***	− 2.612***	− 0.407***	− 0.577***
	(4.77)	(− 16.82)	(− 10.67)	(− 10.89)
$cons$	0.812***	8.660***	10.92***	13.29***
	(461.91)	(19.08)	(98.08)	(85.84)
${R}^{2}$	0.5105	0.6886	0.6149	0.6149
Panel B:$AGG2$				
$AGG2$		3.978***	1.160***	2.674***
		(13.62)	(16.13)	(27.60)
$SCCdid$	− 0.293***	− 1.519***	− 0.101*	0.129*
	(− 34.60)	(− 8.71)	(− 2.36)	(2.23)
$Control$	$TRUE$	$TRUE$	$TRUE$	$TRUE$
$YearFE$	$TRUE$	$TRUE$	$TRUE$	$TRUE$
$City FE$	$TRUE$	$TRUE$	$TRUE$	$TRUE$
$N$	3990	3990	3990	3989
${R}^{2}$	0.571	0.7372	0.6882	0.7821

The values in parentheses refer to the t-values of the regression coefficients; ***, **, and * refer to the significance levels of 1%, 5%, and10%, respectively.
3. Technological effect
  The impact of SCC on PE can be represented using government science and technology policies, which serve as a proxy for technological effects. SCC leverages emerging information technologies to transform traditional industries and organizational management, enabling product upgrades and continuous technological innovation. To examine this mechanism, we test the impact of SCC on government policy on science and technology ($TH$). Specifically, $TH$ is the proportion of regional scientific and technological expenditures to local general public budget expenditures. In Table 12, the coefficient of SCC is positive in column (1), indicating that SCC significantly increases TH. This result suggests that SCC indeed possesses technological effects. Results in columns (2–4) show that when technological effects and $SCCdid$ are both included in the regression equation, SCC construction suppresses urban wastewater and sulfur dioxide emissions through the increase in $TH$. Overall, the results demonstrate that SCC can mitigate PE through technological effects.

**Table 12:** Regression results of the mechanism test for the technological effect.

	(1)	(2)	(3)	(4)
	$lnTH$	$VSD$	$lnEFD$	$lnSO2$
$lnTH$		− 0.210***	0.0548*	− 0.0355*
		(− 3.79)	(2.47)	(− 2.53)
$SCCdid$	0.397***	− 2.485***	− 0.210***	− 0.0732
	(7.14)	(− 15.16)	(− 4.69)	(− 1.58)
$Control$	$TRUE$	$TRUE$	$TRUE$	$TRUE$
$YearFE$	$TRUE$	$TRUE$	$TRUE$	$TRUE$
$City FE$	$TRUE$	$TRUE$	$TRUE$	$TRUE$
$N$	3990	3990	3990	3990

The values in parentheses refer to the t-values of the regression coefficients; ***, **, and * refer to the significance levels of 1%, 5%, and10%, respectively.

## Heterogeneous analysis

### Central cities (CC)

Considering that different cities have different administrative levels, resource endowments, and varying levels of policy and financial support, this study separates provincial CC from non- CC for analysis. As shown in Table 13, SCC in non-CC has an inhibitory effect on VSD in the city. Regardless of whether it is a CC or a non-CC, SCC significantly inhibits EFD and SO_2_ emissions. However, the inhibitory effect on EFD is higher in CC, while the inhibitory effect on SO_2_ emissions is stronger in non-CC. This finding reinforces the idea that as the level of urban development improves, pollution emissions tend to decrease.

**Table 13:** Whether it is Central City heterogeneity analysis regression results.

	(1) non-CC	(2) CC	(3) non-CC	(4) CC	(5) non-CC	(6) CC
	$VSD$	$VSD$	$lnEFD$	$lnEFD$	$lnso2$	$lnso2$
$SCCdid$	− 0.943***	− 0.940	− 0.180***	− 0.372***	− 0.658***	− 0.491**
	(− 4.90)	(− 1.13)	(− 3.87)	(− 3.49)	(− 10.78)	(− 2.83)
$Control$	$TRUE$	$TRUE$	$TRUE$	$TRUE$	$TRUE$	$TRUE$
$YearFE$	$TRUE$	$TRUE$	$TRUE$	$TRUE$	$TRUE$	$TRUE$
$City FE$	$TRUE$	$TRUE$	$TRUE$	$TRUE$	$TRUE$	$TRUE$
$N$	3720	247	3720	247	3720	246
${R}^{2}$	0.7298	0.8025	0.6716	0.7861	0.7746	0.6328

The values in parentheses refer to the t-values of the regression coefficients; ***, **, and * refer to the significance levels of 1%, 5%, and10%, respectively.

### Urban education level

Cities are categorized into two groups based on the presence of "Project 211" universities within the city: those with a high level of education (with "Project 211" universities) and those with a low level of education (without "Project 211" universities). A double difference model is applied. As shown in Table 14, SCC in non-provincial capital cities can suppress VSD, with a higher inhibitory effect in cities with a high level of education and a lower inhibitory effect in cities with a low level of education. SCC also exhibits a significant reduction effect on EFD and SO_2_, with a more pronounced inhibitory effect in cities with lower education levels. The main reason for this is that SCC requires a certain level of acceptance from residents. In areas with higher education levels, there is greater emphasis on talent development, leading to a higher level of acceptance for new initiatives. However, in column 6 of Table 13, an anomaly occurs where, with a high level of education, SCC increases the emissions of SO_2_. This is attributed to the fact that emissions reduction activities for SO_2_ involve changes in both end-of-pipe technologies and production processes^58^. End-of-pipe technologies, such as flue gas desulfurization devices, require a significant amount of low-skilled labor for installation, operation, and maintenance^59^. Existing studies indicate that that enterprises and cities with a higher proportion of low-skilled workers can reduce SO_2_ emissions, SO_2_ intensity, and the overall generation of SO_2_^58–60^. Therefore, in cities with a high level of education, the SCC may lead to a positive effect on SO_2_ emissions. However, as production processes evolve and more efficient and less polluting equipment is installed, the demand for low-skilled labor decreases, subsequently reducing SO_2_ emissions^60^.

**Table 14:** Whether it is high education level cities heterogeneity analysis regression results.

	(1) without	(2) with	(3) without	(4) with	(5) without	(6) with
	$VSD$	$VSD$	$lnEFD$	$lnEFD$	$lnso2$	$lnso2$
$SCCdid$	− 0.652***	− 2.679*	− 0.199***	0.115	− 0.117*	0.845***
	(− 3.79)	(-2.27)	(− 4.33)	(0.73)	(− 2.45)	(4.09)
$Control$	$TRUE$	$TRUE$	$TRUE$	$TRUE$	$TRUE$	$TRUE$
$YearFE$	$TRUE$	$TRUE$	$TRUE$	$TRUE$	$TRUE$	$TRUE$
$City FE$	$TRUE$	$TRUE$	$TRUE$	$TRUE$	$TRUE$	$TRUE$
$N$	3700	266	3700	266	3700	265
${R}^{2}$	0.8029	0.7293	0.6996	0.7020	0.7867	0.7340

The values in parentheses refer to the t-values of the regression coefficients; ***, **, and * refer to the significance levels of 1%, 5%, and10%, respectively.

### Urban scale

The classification of city sizes in this paper is based on the latest standards set by the Chinese State Council in 2014. Since there are fewer small-scale cities, which makes the research results unreliable, only the results for cities of type ii and above are reported.

Table 15 indicate that SCC can significantly mitigate PE, but the significance varies in cities of different scales. Large-scale cities exhibit the highest inhibitory effect. Medium-scale cities also show significant reduction in EFD, with a relatively high level of significance. For cities of Type II scale and above, SCC restrains VSD and SO2 emissions, and the larger the city scale, the greater the inhibitory effect. This suggests that larger cities have increased resource utilization efficiency, which is more conducive to environmental protection. This conclusion underscores that when urban development patterns transform and innovation deepens, pollution issues from traditional urban development processes can be addressed. The key to pollution control is not the size of the city but the renewal of urban governance models and technological advancements.

**Table 15:** Whether it is Urban Scale heterogeneity analysis regression results.

	(1) Type ii cities	(2) Medium-scale cities	(3) Large-scale cities	(4) Super-large-scale cities
Panel A:$VSD$				
$SCCdid$	− 0.300	− 0.401	− 1.617***	− 2.546
	(− 0.80)	(− 1.71)	(− 4.77)	(− 1.63)
${R}^{2}$	0.7977	0.8672	0.9437	0.7374
Panel B:$lnEFD$				
$SCCdid$	0.407	− 0.194***	− 0.225**	0.0227
	(1.49)	(− 3.53)	(− 2.66)	(0.12)
${R}^{2}$	0.8584	0.7032	0.6802	0.9211
Panel C:$lnso2$				
$SCCdid$	0.795	0.0221	− 0.262**	− 0.0117
	(1.72)	(0.38)	(− 2.80)	(− 0.04)
$Control$	$TRUE$	$TRUE$	$TRUE$	$TRUE$
$$ YearFE$$	$$TRUE$$	$$TRUE$$	$$TRUE$$	$$TRUE$$
$$ City FE$$	$$TRUE$$	$$TRUE$$	$$TRUE$$	$$TRUE$$
$N$	134	2618	1086	117
$$ {R}^{2}$$	0.8428	0.7935	0.7900	0.9007

The values in parentheses refer to the t-values of the regression coefficients; ***, **, and * refer to the significance levels of 1%, 5%, and10%, respectively.

## Conclusions and policy recommendations

### Conclusions

SCC not only leads the application of information technology but also has a significant impact on enhancing the overall competitiveness of cities. In this study, SCC is considered a "quasi-natural experiment," and based on data from 210 prefecture-level cities in China from 2003 to 2021, a Time-Varying DID model is employed to elucidate the impact of SCC on PE. Simultaneously, the study explores the mechanism pathways through which SCC reduces PE from the perspectives of scale effects, structural effects, and technological effects. Finally, heterogeneity analysis is conducted based on central cities, education levels, and city sizes. The main research findings are as follows:

1. After a series of robustness tests, such as parallel trend tests, placebo tests, PSM-DID, etc., the results consistently indicate that SCC has a certain degree of negative impact on PE. In comparison to non-smart cities, smart cities significantly reduce VSD by approximately 95.3%, decrease SO_2_ by about 11.3%, and reduce EFD by approximately 18.0%.
2. Mechanism analysis results demonstrate that SCC mitigates urban environmental pollution by suppressing the scale effects related to population and industrial production, thus adjusting the industrial structure and promoting the shift towards the service sector. Additionally, SCC mitigates environmental pollution through technological effects, with a more pronounced inhibitory effect on VSD. These findings provide insights into the effectiveness of SCC in reducing urban PE and highlight the importance of considering heterogeneity and mechanisms in the evaluation of policy impacts.
3. Heterogeneity analysis reveals that the impact of SCC on urban PE varies. CC exhibit a higher inhibitory effect on PE compared to non-CC. Cities with a high level of education experience greater inhibitory effects on PE, while those with a lower level of education experience weaker effects. Larger cities have the highest inhibitory effect on PE, with the inhibitory effect increasing as the city scale becomes larger.

### Policy recommendations

This paper provides a meso-level analysis from the perspective of prefecture-level cities, exploring the inhibitory effect of SCC on PE and delving into the underlying mechanisms. Based on the research conclusions, the following three policy recommendations are proposed to further address environmental pollution issues in China:

Firstly, in order to effectively reduce urban PE and mitigate environmental pollution problems, the Chinese government should elevate technological research and development innovation to a strategic position within the framework of SCC. This involves focusing on the organization of research teams and the establishment of platforms, creating a conducive research environment. Simultaneously, increasing investment in technological research and development funding and reinforcing financial support is essential. Collaborating with outstanding talents from universities and research institutes will provide human resources for research and innovation, leveraging the foundational support role of technological innovation in SCC.

Secondly, leveraging SCC to harness the pollution reduction effects of structural, scale, and technological effects. Building on technological progress, promote the transformation of urban industries from labor-intensive to technology-intensive. Utilize smart industries to achieve the transformation and upgrading of urban industrial structures, with a specific focus on supporting the development of low-energy and environmentally friendly industries. Unleash the pollution reduction effects of structural and technological aspects within smart cities.

Thirdly, capitalize on the radiating effects of central cities, large-scale cities, and cities with high levels of education on surrounding areas. Through institutional and mechanism innovation, establish secure and effective mechanisms for data governance and development. Unlock the vitality of urban data elements, extending the advanced governance capabilities of central cities to the entire region. Strengthen government governance capabilities comprehensively in both vertical and horizontal directions through innovative institutional mechanisms for data utilization.

## Data Availability

The data that support the findings of this study are available from [https://data.stats.gov.cn/] but restrictions apply to the availability of these data, which are used under license for the current study, and so are not publicly available. Data are however available from the authors upon reasonable request and with permission of [https://data.stats.gov.cn/]. Those wishing to request data from this study can contact GuoWei Zhang.
